# The Correlation between Objective Ligament Laxity and the Clinical Outcome of Mechanically Aligned TKA

**DOI:** 10.3390/jcm12186007

**Published:** 2023-09-16

**Authors:** Stefano Campi, Rocco Papalia, Carlo Esposito, Vincenzo Candela, Andrea Gambineri, Umile Giuseppe Longo

**Affiliations:** 1 Orthopaedic and Trauma Surgery, Fondazione Policlinico Universitario Campus Bio-Medico, Via Alvaro del Portillo 200, 00128 Rome, Italy; s.campi@policlinicocampus.it (S.C.); r.papalia@policlinicocampus.it (R.P.); v.candela@policlinicocampus.it (V.C.); 2 Research Unit of Orthopaedic and Trauma Surgery, Department of Medicine and Surgery, Università Campus Bio-Medico di Roma, Via Alvaro del Portillo 21, 00128 Rome, Italy; c.esposito@unicampus.it (C.E.); a.gambineri@unicampus.it (A.G.)

**Keywords:** knee, arthroplasty, instability, of medial laxity, lateral laxity

## Abstract

Instability is one of the causes of failure in total knee arthroplasty (TKA). The aim of this study was to analyze the correlation between objective ligament laxity and the clinical outcome of mechanically aligned TKA. Fifty-one knees in 47 patients were evaluated at a minimum follow-up of 6 months. The correlation between the angular displacement and functional scores (Knee Society Score and Knee Injury and Osteoarthritis Score) was analyzed. A negative correlation (*p*-value < 0.05) was observed between medial laxity ≥5° at 0, 30, 60, and 90° of flexion and the outcome measures. Lateral laxity did not correlate with the clinical outcome. At 30° of knee flexion, a total varus and valgus laxity ≥10° was related to poorer outcomes. The same amount of angular displacement did not influence the outcome in the other flexion angles. There was no difference in single-radius vs multi-radius implants in terms of medial and lateral laxity and clinical outcome. A valgus displacement ≥5° measured at 0, 30, 60, and 90 degrees of flexion correlated with an inferior clinical outcome. In contrast, the same amount of displacement measured on the lateral compartment did not influence the clinical outcome after TKA.

## 1. Introduction

Instability following primary total knee arthroplasty (TKA) is one of the major failure mechanisms leading to revision surgery [1]. However, the difference between “physiological” and “pathological” ligament laxity after mechanically aligned TKA remains unclear. Soft-tissue balancing is critical for successful TKA, providing stability and driving knee kinematics. However, the ideal range of medial and lateral ligament laxity of mechanically aligned TKA remains unclear, mainly because of the difficulty in achieving reliable measurements and the high heterogeneity among individuals.

In TKA practice, surgeons assess knee laxity both intraoperatively and at follow-up. Ligament balancing is based on bone resection and soft-tissue management, on patient’s native phenotype and deformity, and implant design.

Different surgical techniques for ligament balancing have been developed [2,3], but in most cases soft-tissue balance is not based on an objective evaluation, depending mainly on surgeons’ experience and preferences. Furthermore, the ligament laxity assessed with trial implants may vary when compared to the final implant [4] and change over time after the operation.

Several methods for measuring knee laxity during the clinical evaluation are available, but a gold standard has not yet been established.

Measuring medial and lateral laxity in the operating room throughout the range of motion is very difficult using conventional instruments. New technologies, such as navigation and robotic devices, have allowed to objectively measure tibio-femoral gaps in real time and to provide a better understanding of the effects of implant alignment on joint laxity before bone resections. However, these technologies are available but not incorporated in clinical practice on a large scale.

Medial, lateral, and sagittal joint stability have been reported to influence postoperative outcome [5,6]. However, the correlation between coronal and sagittal ligament laxity and patient-reported outcomes is controversial [7,8]. Only few studies have evaluated both the coronal and sagittal ligament laxity [9,10,11].

Similarly, studies on the influence of the curvature radius of the femoral component on mid-flexion stability have proved to be contradictory. Several studies have shown increased stability at 30° degrees of flexion in single-radius (SR) prostheses without, however, significant differences in outcomes between single-radius vs multi-radius groups [12]. Other studies have found no significant differences in varus–valgus stability between multi-radius (MR) and SRimplants, suggesting that the instability may be the result of unrecognized ligament laxity or technical errors during surgery rather than a factor intrinsic to the prosthetic implant [13].

The aim of our study is to investigate (1) the relationship between laxity in varus and valgus stress at 0°, 30°, 60°, and 90° knee flexion, anteroposterior translation measured at 90°, and the clinical outcome scores (Knee Injury and Osteoarthritis Outcome Score–KOOS and Knee Society Score–KSS); (2) the correlation between the use of single or multi-radius implants, laxity in varus–valgus, and clinical outcome.

## 2. Materials and Methods

A prospective evaluation of patients who underwent TKA surgery at the Campus Bio-Medico Hospital in Rome between October 2019 and July 2021 was performed. Data of the enrolled patients were subsequently collected between May 2021 and September 2021.

### 2.1. Patients’ Selection

Patients who underwent primary TKA with minimum 6-month follow-up were considered eligible for the study.

Inclusion criteria were patients who underwent primary TKA at Campus Bio-Medico Hospital of Rome, minimum follow-up of 6 months, absence of intraoperative complications, absence of preoperative varus deformity >20°, absence of preoperative valgus deformity >15°, absence of preoperative flexion deformity >20°, absence of previous surgery, and infection of fractures on the knee.

Exclusion criteria were presence of a semi-constrained or constrained prosthetic implant, preoperative varus deformity >20° and valgus >15°, preoperative flexion deformity >20°, ligamentous or intraoperative iatrogenic tendon injuries, intraoperative fractures, previous tibial or femoral osteotomy, previous knee fractures, severe extra-articular deformities, inflammatory and autoimmune rheumatological diseases, history of previous prosthetic infection, neuropathies, and neuromuscular pathologies.

An experienced orthopedic knee surgeon who had more than 10 years of experience in knee surgery examined participants for inclusion and exclusion. To avoid selection bias and errors, included patients were then assessed by the Senior Author.

### 2.2. Surgical Technique

All procedures were performed through a central skin incision and a medial parapatellar arthrotomy. The anterior cruciate ligament was removed in all cases, while the posterior cruciate ligament was preserved or resected based on the type of prosthetic implant used. The type of prosthetic alignment performed is mechanical alignment. The distal femoral cut was made perpendicular to its mechanical axis in the coronal plane as measured on preoperative standing hip–knee–ankle (HKA) radiographs with the use of an intramedullary guide. The proximal tibial cut was then performed perpendicular to its mechanical axis in the coronal plane and with a 3–7° posterior tibial slope in the sagittal plane using extramedullary guide. Verification of the correct balance of the femoral and tibial cuts was carried out in extension. In order to reach the correct balance in extension, after selecting the correct size of the femoral component with an anterior or posterior reference system, the oblique, posterior condylar, and anterior cortical cuts of the femur were performed. Rotation of the femoral component was established by drawing the transepicondylar axis and the Whiteside line with 3–5° external rotation from the posterior condylar line.

Ligament releases were performed to achieve adequate balance. The prosthetic components were fixed without (6 knees) and with cementation (45 knees). Patella prosthesis was performed in eight cases. All surgeries were performed with tourniquet insufflation. Full weight bearing, quadriceps muscle setting, and range of motion exercises were started the day after surgery.

### 2.3. Laxity Measurements

**Figure 1:**
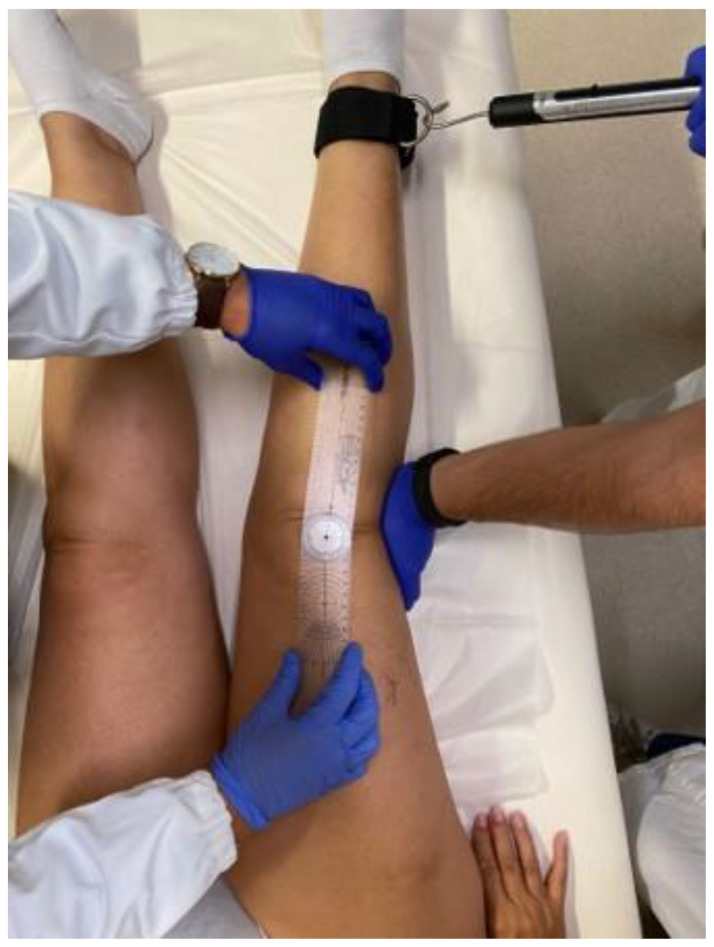
Valgus stress performed in full extension applying a standard force of 10 kg through the use of a dynamometer.

**Figure 2:**
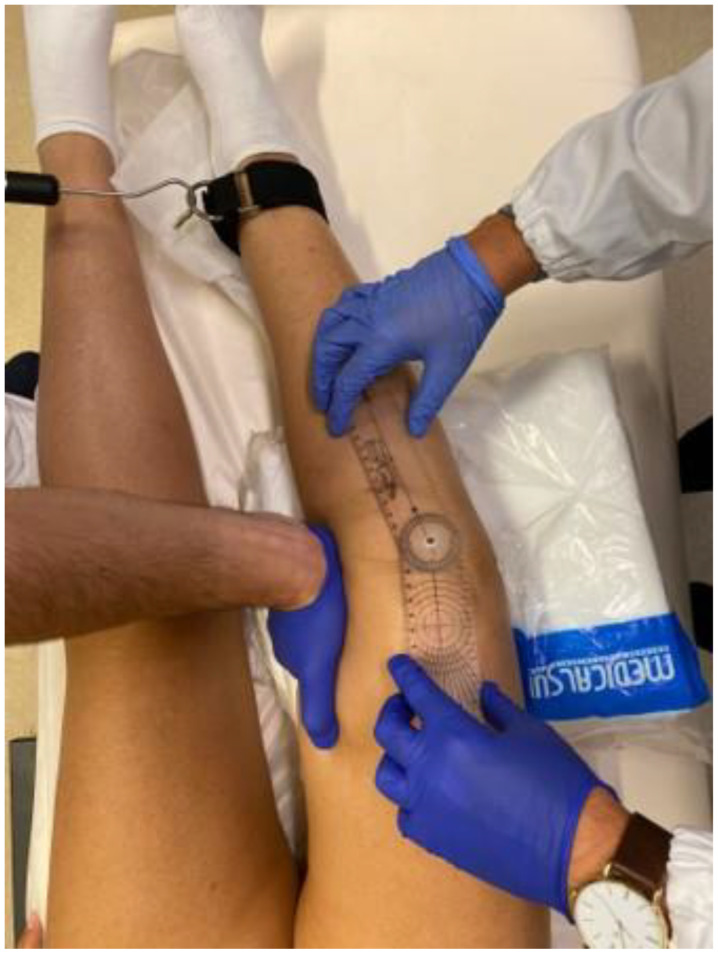
Varus stress performed at 30° of knee flexion applying a standard force of 10 kg through the use of a dynamometer.

Knee laxity was clinically evaluated both in the coronal and sagittal planes. To assess coronal laxity, a varus stress and a valgus stress were applied in full extension (Figure 1: valgus stress performed in full extension applying a standard force of 10 kg through the use of a dynamometer) and 30° (Figure 2: varus stress performed at 30° of knee flexion applying a standard force of 10 kg through the use of a dynamometer), 60°, and 90° knee flexion, applying a standard force of 10 kg through the use of a dynamometer (Salter Little Samson Dynamometer, Brecknell Fairmont, MN 56031-1439 USA) attached to an ankle in order to reduce the rotational forces that could have affected the results. The degree of opening in varus and valgus stress was measured clinically with an orthopedic goniometer [14].

**Figure 3:**
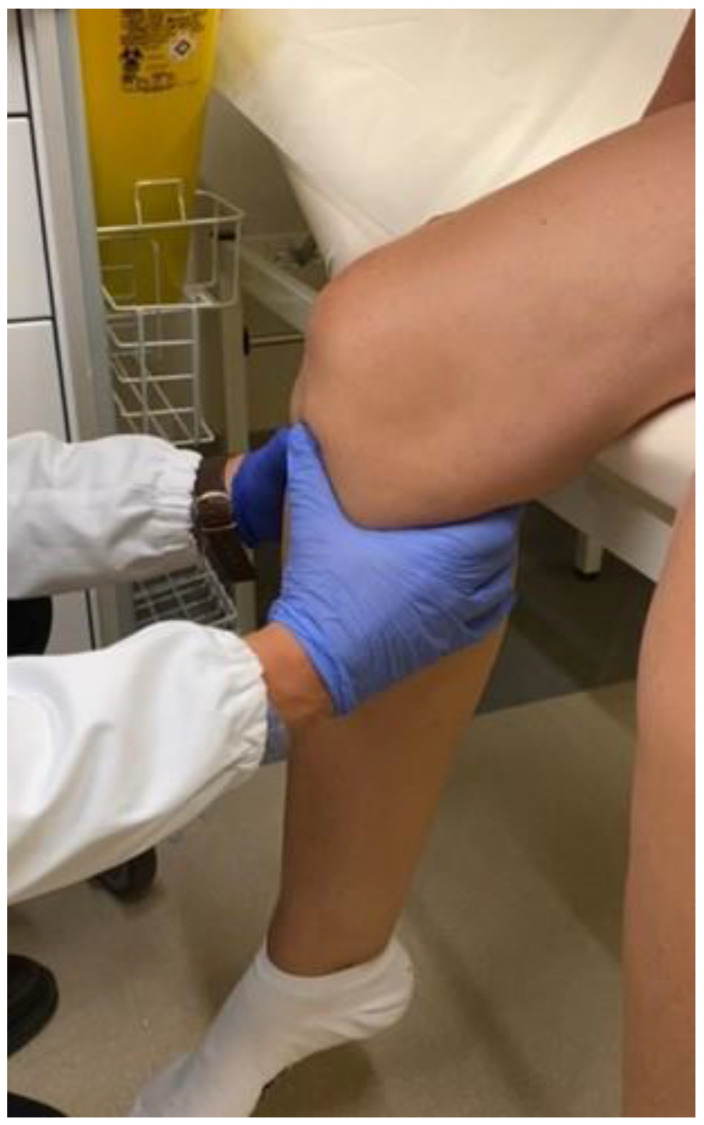
Sagittal laxity at 90° knee flexion measured with the drawer test performed with the knee flexed at 90° with the quadriceps relaxed and the foot free.

The ROM (range of motion) was also measured with a goniometer (Shahe, China) [15]. The sagittal laxity at 90° knee flexion was measured with the drawer test (Figure 3: sagittal laxity at 90° knee flexion measured with the drawer test performed with the knee flexed at 90° with the quadriceps relaxed and the foot free), performed with the knee flexed at 90° with the quadriceps relaxed and the foot free [16,17,18].

The Intraobserver reliability of the testing procedure was assessed in a preliminary study. In this preliminary study on 10 patients, the same test was performed twice by the same orthopedic knee surgeons. Intraobserver reliability was 0.83.

### 2.4. Clinical Outcome

The evaluation of clinical outcomes was carried out with the Knee Injury and Osteoarthritis Outcome Score (KOOS) [19,20] and the Knee Society Clinical Rating System (KSS) [21] at minimum follow-up of 6 months follow-up.

KOOS is a knee-specific subjective questionnaire consisting of forty-two questions, divided into five sections: subscales for pain, other symptoms and stiffness, activities of daily living (ADLs), function in sport and recreation, and knee-related quality of life (QOL). The KSS consists of two sections, “Knee Score” (KSS) and “Functional Score” (KSS-F), and provides us with an objective assessment of the functional prosthetic outcome. The self-administered questionnaires were completed by the patient alone.

### 2.5. Statistical Analysis

Data were summarized using mean and standard deviation (Mean ± SD). The normal distribution of the variables was verified by means of the Shapiro–Wilk test. Spearman’s correlation was used to evaluate the correlation between laxity and scores. The Mann–Whitney U Test was used to evaluate statistically significant differences between total laxity (varus + valgus) <10° vs. ≥10°, varus laxity <5° vs. ≥5°, valgus laxity <5° vs. ≥5°, anteroposterior translation “<5 mm” vs. “≥5 mm” group, and “Single radius” group vs. “multi-radius” group in the various scores. The level of statistical significance was set *p* < 0.05. Correlation values: <0.3 low; [0.3–0.39] moderate; [0.4–0.69] high; >0.70 very strong. The post hoc power analysis made by using G power 3.1 for the correlation between medial laxity and KKS showed that the power of the study is 0.8 for a mean correlation (r), 0.38 for an alpha value of 0.05, and a sample size of 51.

## 3. Results

This study included 51 knees (20 right knees and 31 left knees) in a total of 47 patients (31 females and 16 males) with a mean age of 69.6 ± 8.3 years. Clinical outcomes were assessed at a mean follow-up of 7.2 months (SD 2.64, range 6–18).

A single-radius TKA was used in 19 cases and a multi-radius in 32 cases. The following implants were used in the single-radius group: GMK^®^ Sphere Medacta, Triathlon^®^ CR Stryker, Triathlon^®^ PS Stryker, and Triathlon^®^ CS (cruciate-substituting) Stryker. The following implants were used in the multi-radius group: Persona^®^ PS Zimmer-Biomet, Persona^®^ MC (medial congruent bearing) Zimmer-Biomet, Nexgen^®^ PS Zimmer-Biomet, and Journey^®^ II CR Smith & Nephew (Table 1).

The mean lateral and medial laxity measurement is reported in Table 2.

### 3.1. Coronal Laxity and Clinical Outcomes

There was a significant negative correlation between medial laxity at 0° and KOOS (r −0.304, *p* 0.03), K-Symptoms and Stiffness (r −0.43, *p* 0.002), K-Pain (r 0.29, *p* 0.04), K-Quality of Life (r −0.34, *p* 0.01), KSS (r −0.33, *p* 0.02), and KSS-Function (r −0.47, *p* < 0.001).

A high negative correlation was observed between increased medial laxity at 30° of flexion and KOOS (r −0.502, *p* < 0.001), K-Symptoms and Stiffness (r −0.415, *p* 0.002), K-Sports (r −0.415, *p* 0.002), K-Function Daily Living (r −0.407, *p* 0.003), K-Quality of Life (r −0.471, *p* < 0.001), KSS (r −0.455, *p* 0.001), and KSS-f (r −0.521, *p* < 0.001).

At 60° of flexion, we found a low negative correlation between medial laxity and KOOS (r −0.286, *p* 0.042) and K-Quality of Life (r −0.298, *p* 0.034) and a moderate negative correlation between medial laxity and K-Symptoms and Stiffness (r −0.365, *p* 0.008), KSS (r −0.306, *p* 0.029), and KSS-F (r −0.364, *p* 0.009).

Finally, medial laxity at 90° of flexion showed a low negative correlation with KOOS (r −0.294, *p* 0.036) and KSS (r −0.299, *p* 0.033) and a moderate negative correlation with K-Symptoms and Stiffness (r −0.349, *p* 0.012), K-Quality of Life (r −0.325, *p* 0.020), and KSS-F (r −0.337, *p* 0.016).

Lateral laxity showed no significant correlation with the reported outcome measures at 0°, 30°, 60°, and 90° of flexion, with some minor exceptions: a low positive correlation with K-Pain at 30° of flexion (r 0.289, *p* 0.040), a moderate positive correlation with ROM Max at 30° of flexion (r 0.305, *p* 0.029), a moderate positive correlation with K-Pain (rho = 0.303, *p* = 0.031), KSS-F (rho = 0.307, *p* = 0.028), and ROM Max (rho = 0.311, *p* = 0.026) at 60° flexion (Table 3).

Patients with a medial laxity greater that 5° at 0, 30, 60, and 90 degrees of flexion reported significantly lower outcome measures when compared with patients with less than 5° of laxity (Table 4).

There was no significant difference between patients with a lateral laxity greater than 5° versus lower than 5° at any flexion degree (Table 5).

Patients with more than 10° of overall laxity (medial + lateral) showed a statistically significant decrease in the KOOS score, K-Symptoms and Stiffness, K-Pain, K-Function daily living, K-sports, K-quality of life, KSS, and KSS-F at 30° of flexion when compared with patients with less than 10° of overall laxity (Table 6).

### 3.2. Sagittal Laxity and Clinical Outcomes

No statistically significant differences in clinical outcome were found between patients with an anteroposterior translation <5 mm or >5 mm at 90° flexion (Table 7).

### 3.3. Single-Radius vs. Multi-Radius Implant

No statistically significant differences were found in terms of clinical outcomes and coronal laxity measured at 30°, 60°, and 90° between single-radius and multi-radius implants (Table 8).

## 4. Discussion

The results of this study suggest that an increase in medial laxity at 0, 30, 60, and 90 degrees of flexion is correlated with poorer postoperative outcome of mechanically aligned TKA. Lateral laxity does not affect the clinical scores. There was no difference in the incidence of postoperative laxity and in the clinical outcome between single-radius vs. multi-radius implants.

These results are in line with previous studies demonstrating that medial stability is essential for an adequate functioning of the implant, while a lateral laxity does not negatively affect the clinical outcome. Indeed, a medial laxity induces non-physiological kinematics of the knee, while a lateral laxity has little effect on the kinematics [22,23] being physiological in the native knee both in extension and flexion. Okazaki et al. [24] analyzed 50 healthy knees with varus–valgus stress radiographs in extension and flexion and reported the following mean coronal laxity values as physiological: 4.9° and 2.4° of lateral and medial laxity in extension and 4.8° and 1.7° of lateral and medial flexion laxity, respectively.

Previous studies reported similar results. Tsukiyama et al. [6] found that knees with medial joint laxity during flexion resulted in an inferior postoperative outcome, while lateral joint laxity did not influence patient satisfaction or function. Aunan et al. [25] analyzed the association between ligamentous laxity measured intraoperatively and clinical outcome at one year of follow-up in 108 patients with TKR. Medial and lateral laxity were measured in extension and 90° knee flexion. They found a worsening of postoperative pain and knee function directly proportional to the increase in medial laxity both in extension and 90° of flexion. Watanabe et al. [11] found that lateral laxity was greater than the medial one both in extension and at 80° of flexion in all knees; the value of 3.6° was also defined as the ideal value of medial laxity in extension and at 80° of flexion, with a worsening of the overall satisfaction and pain scores due to increases in medial laxity above this threshold. Tanaka et al. [26] found that an asymmetrical coronal balance in extension and 90° knee flexion has no effect on postoperative ROM and on the subscales of the modified KSS and that a relative increase in lateral laxity does not lead to a worsening of clinical symptoms and function of the operated knees. Nakano et al. [27] reported an increase in ROM Max with increasing lateral laxity measured at 90° knee flexion. Seah et al. [28] evaluated the relationship between coronal stability measured at 30° knee flexion and clinical outcome. Better scores were associated with total laxity (varus + valgus) <5°. Matsuda et al. [29] studied the overall effects of varus–valgus laxity measured exclusively in extension on the ROM Max at one year of follow-up. The results obtained showed a significant increase in ROM in patients with a difference in laxity <2° and a concomitant increase in ROM in patients with a total laxity (varus + valgus) between 6–10°. Similar results were obtained by Yoshihara et al. [30] who analyzed coronal laxity in extension and 90° knee flexion. The results obtained identified as acceptable coronal laxity values <5° in valgus or varus and determined that a total laxity <10 ° in both extension and flexion did not determine either a worsening of the clinical outcome, calculated with KSS, or an increase in the prosthetic failure rate.

In the present study, the sagittal laxity at 90° knee flexion was evaluated and correlated with the clinical outcome. No statistically significant differences in clinical scores were found between patients with values <5 mm and values >5 mm. However, all patients had translational values <10 mm.

Jones et al. [31] observed in 97 knees undergoing CR TKR a decrease in maximum ROM and KSS in patients with AP translation >10 mm at 75° knee flexion compared to patients with an AP translation between 5–10 mm, concluding that the latter was the optimal range of sagittal stability. Watanabe et al. [11] indicated that adequate values of AP translation measured at 75° knee flexion were those in the range 5–10 mm. Warren et al. [32] in a comparative study on sagittal laxity among PS, CR, and double cruciate retention prostheses observed an increase in ROM max in patients with AP translation >5 mm, regardless of the type of prosthetic implant used, but did not identify a pathological upper limit of translation. Matsumoto et al. [33,34], evaluated in 110 knees undergoing PS TKR the association between sagittal laxity at 30°, 60°, and 90° knee flexion and functional outcomes and observed a significant decrease in the K-pain score with increasing AP translation at 60° knee flexion.

Finally, no statistically significant differences emerged regarding clinical outcomes and coronal laxity measured at 0, 30, 60, and 90 degrees of flexion between groups single-radius vs multi-radius implants.

Some studies have shown that the transition from a longer to a shorter radius in MR prostheses causes temporary instability during knee flexion between 30° and 45° due to a probable loss of tension in the collateral ligaments [35,36,37,38]. In contrast, some studies have shown increased stability at 30° of flexion in SR prostheses without, however, significant differences in outcomes between the two groups [12]. Other studies have found no significant differences in varus–valgus stability between MR and SR implants, suggesting that the instability may be the result of unrecognized ligament laxity during surgery rather than a factor dependent on intrinsic characteristics of the implant [13,39,40].

Several limitations of this study should be acknowledged. The main limitation concerns the method of measuring laxity. Both coronal laxity and sagittal laxity were measured clinically through the use of a dynamometer and a goniometer for the assessment of medial-lateral laxity in varus–valgus stress. The evaluation of sagittal laxity was conducted with the execution of the drawer test. The choice of the goniometer and dynamometer has proven to be more reliable from the comparison with the current literature: at present, there is no instrument that allows to measure laxity precisely in a clinical setting. Since the measurements were only taken clinically, it was equally difficult to accurately identify and distinguish the subtle changes in degrees of laxity. The choice to perform radiographic measurements of the degree of laxity, as was conducted by some of the studies cited in the text, would have significantly improved the significance of our results. However, the difficulty in carrying out further radiographic investigations must be taken into consideration, both at a more strictly hospital level and due to the poor compliance of the patients enrolled. The radiographic investigation flanked by advanced computer-assisted navigation systems, similar to those already used in surgical practice, could further improve the overall assessment of instabilities [41,42].

The number of patients enrolled in our study is relatively small when compared with similar studies in the literature. We tried to include only patients undergoing more recent prosthetic implants in the study to ensure that the surgical technique and postoperative rehabilitation did not differ significantly.

We were unable to calculate the preoperative laxity because it is not possible to objectively quantify the preoperative medial or lateral bone loss and how it can affect ligamentous stability. A possible significant correlation between preoperative and postoperative gap balances, both in extension and flexion, could influence our results. This aspect could be the subject of future studies.

In our study, we did not check if the medial-lateral laxity measured for the CR- and PS-TKAs revealed statistically significant differences over the studied flexion arc for the two versions of TKA. Excision of the PCL results in an increased flexion gap, which should increase varus–valgus laxity of the knee with increasing flexion. Our results could be influenced by the heterogeneity of the experimental group.

Moreover, clinical laxity was assessed by a single experienced orthopedic knee surgeon. The Intraobserver reliability of the testing procedure was assessed in a preliminary study to guarantee the accuracy of the measurement. However, the interobserver reliability was not assessed. Although a reproducible method was used for clinical measurements, the absence of an interobserver reliability evaluation could be a limitation of the study.

Finally, the selected patients were not stratified by homogeneous classes of preoperative deformity (varus and valgus) with consequent differences in the preoperative ligament structure [43,44].

Patient satisfaction after TKA is generally lower than after total hip arthroplasty. Several preoperative and intraoperative factors could affect the postoperative outcome. Among these factors, ligament balance could be associated with the subjective and functional results of patients. Few studies have analyzed the relationship between ligament balancing and patient-reported outcomes. The results of this study suggest that an increase in medial laxity at 0, 30, 60, and 90 degrees of flexion is correlated with poorer postoperative outcome of mechanically aligned TKA. These data could drive surgeons to focus on the relevance of medial stability of total knee arthroplasty. Future research is needed with the greatest and more homogeneous populations to obtain high-quality evidence on this topic.

## 5. Conclusions

The results of this study suggest that an increase in medial laxity at 0, 30, 60, and 90 degrees of flexion is correlated with poorer postoperative outcome of mechanically aligned TKA, while lateral laxity does not affect the clinical scores. An overall laxity (medial + lateral) of more than >10° at 30° of flexion leads to a lower clinical outcome. There was no difference in the incidence of postoperative laxity and in the clinical outcome between single-radius vs. multi-radius implants. Finally, an anteroposterior translation lower than 10 mm at 90 degrees of flexion does not influence the results of TKA.

## Figures and Tables

**Table 1:** Single-radius and multi-radius implants and relative number.

Single-Radius	*n* 19	Multi-Radius	*n* 32
GMK Sphere	6	Persona PS	19
Triathlon CR	8	Persona MC	10
Thriatlon PS	3	Nexgen PS	1
Thriatlon CS	2	Journey II CR	2

**Table 2:** Mean medial and lateral laxity (SD) at °, 30°, 60°, and 90° of knee flexion.

	Lateral Laxity (°)	Medial Laxity (°)
0°	1.5 (0.83)	1.6 (1.02)
30°	3.6 (1.82)	2.9 (2.14)
60°	4.6 (2.41)	3.6 (2.36)
90°	3.3 (1.81)	2.9 (2.19)

**Table 3:** Correlation between coronary laxity at 0°, 30°, 60°, and 90° knee flexion and clinical outcomes.

			KOOS	K-Symptoms and Stifness	K-Pain	K-Function Daily Living	K-Sports	K-Quality of Life	KSS	KSS-F	ROM Max
0°	LATERAL	rho	−0.185	−0.294	−0.129	−0.137	−0.001	−0.128	−0.251	−0.076	0.119
		P	0.194	0.036	0.365	0.338	0.993	0.369	0.075	0.598	0.406
	MEDIAL	rho	−0.304	−0.425	−0.287	−0.171	−0.161	−0.344	−0.335	−0.471	−0.263
		P	0.030	0.002	0.041	0.229	0.259	0.013	0.016	<0.001	0.062
30°	LATERAL	rho	0.144	0.134	0.289	0.083	0.006	0.174	0.114	0.241	0.305
		P	0.312	0.348	0.040	0.562	0.964	0.222	0.427	0.088	0.029
	MEDIAL	rho	−0.502	−0.415	−0.391	−0.407	−0.415	−0.471	−0.455	−0.521	−0.168
		P	0.000	0.002	0.005	0.003	0.002	0.000	0.001	0.000	0.240
60°	LATERAL	rho	0.145	0.214	0.303	0.085	−0.008	0.158	0.062	0.307	0.311
		P	0.309	0.131	0.031	0.555	0.957	0.267	0.667	0.028	0.026
	MEDIAL	rho	−0.286	−0.365	−0.256	−0.203	−0.194	−0.298	−0.306	−0.364	−0.179
		P	0.042	0.008	0.070	0.153	0.172	0.034	0.029	0.009	0.209
90°	LATERAL	rho	0.002	0.101	0.086	−0.015	−0.084	0.030	−0.084	0.186	0.242
		P	0.991	0.480	0.548	0.915	0.558	0.836	0.558	0.191	0.087
	MEDIAL	rho	−0.294	−0.349	−0.249	−0.229	−0.258	−0.325	−0.299	−0.337	−0.179
		P	0.036	0.012	0.078	0.106	0.068	0.020	0.033	0.016	0.209

**Table 4:** Comparison of clinical outcome for patients with a medial laxity >5° versus patients with medial laxity <5°.

		<5°	>5°	*p*-Value
0°	Knees	0	0	
30°	Knees	40	11	
	KOOS	77.67 ± 11.91	55.55 ± 18.12	0.001
	KSS	88.83 ± 8.16	73.82 ± 13.14	0.001
	KSS-F	81.75 ± 15.17	57.73 ± 9.32	<0.001
60°	Knees	34	17	
	KOOS	78.42 ± 11.42	61.84 ± 18.72	0.002
	KSS	88.59 ± 8.62	79.59 ± 13.45	0.013
	KSS-F	82.65 ± 14.63	64.41 ± 15.80	<0.001
90°	Knees	38	13	
	KOOS	78.11 ± 11.65	57.67 ± 18.18	0.001
	KSS	88.95 ± 8.29	75.77 ± 13.03	0.001
	KSS-F	81.58 ± 14.43	61.92 ± 16.78	<0.001

**Table 5:** Comparison of clinical outcome for patients with a lateral laxity >5° versus patients with lateral laxity <5°. (NS: not significant).

		<5°	>5°	*p*-Value
0°	Knees	0	0	
30°	Knees	36	15	
	KOOS	72.33 ± 15.59	74.25 ± 17.90	NS
	KSS	85.47 ± 11.36	85.87 ± 11.16	NS
	KSS-F	74.58 ± 17.38	81.33 ± 16.42	NS
60°	Knees	22	29	
	KOOS	72.10 ± 17.89	73.50 ± 14.99	NS
	KSS	84.77 ± 11.06	86.21 ± 11.45	NS
	KSS-F	71.59 ± 19.72	80.34 ± 14.26	NS
90°	Knees	36	15	
	KOOS	72.81 ± 17.56	73.10 ± 12.65	NS
	KSS	85.69 ± 10.96	85.33 ± 12.13	NS
	KSS-F	74.86 ± 18.34	80.67 ± 13.87	NS

**Table 6:** Comparison of clinical outcome for patients with an overall laxity >10° versus <10°.

		<10	>10	*p*-Value
0°	Knees	0	0	
30°	Knees	46	5	
	KOOS	74.96 ± 15.21	53.94 ± 12.56	0.005
	KSS	86.91 ± 10.14	73.40 ± 14.28	0.015
	KSS-F	78.15 ± 17.24	62.00 ± 8.37	0.038
60°	Knees	34	17	
	KOOS	73.95 ± 15.68	70.79 ± 17.33	0.562
	KSS	87.03 ± 10.65	82.71 ± 12.01	0.141
	KSS-F	79.12 ± 16.21	71.47 ± 18.52	0.144
90°	Knees	43	8	
	KOOS	74.64 ± 14.94	63.50 ± 20.09	0.125
	KSS	86.53 ± 10.75	80.50 ± 12.91	0.139
	KSS-F	78.95 ± 16.39	63.75 ± 16.85	0.021

**Table 7:** Comparison of patients with antero-posterior translation >5 mm vs. <5 mm at 90° of flexion.

	<5 mm (*n* = 34)	>5 mm (*n* = 17)	*p*-Value
KOOS	73.45 ± 16.57	71.79 ± 15.69	0.660
K-Symptoms and Stiffness	75.42 ± 17.56	76.26 ± 15.57	0.992
K-Pain	79.09 ± 18.24	79.58 ± 13.64	0.771
K-Function Daily Living	80.80 ± 16.86	77.86 ± 15.32	0.395
K-Sports	43.97 ± 28.39	38.53 ± 30.76	0.609
K-Quality of Life	60.48 ± 25.41	63.97 ± 24.76	0.630
KSS	86.38 ± 10.44	84.00 ± 12.75	0.603
KSS-F	76.32 ± 17.29	77.06 ± 17.59	0.879
ROM Max	112.5 ± 7.2	114.4 ± 10.6	0.162

**Table 8:** Comparison of single-radius vs multi-radius implants.

	Single Radius (*n* = 19)	Multradius (*n* = 32)	*p*-Value
KOOS	71.08 ± 13.80	73.97 ± 17.51	0.424
K-Symptoms and Stiffness	72.18 ± 16.13	77.78 ± 17.04	0.138
K-Pain	76.32 ± 15.83	80.99 ± 17.21	0.171
K-Function Daily Living	79.64 ± 13.76	79.92 ± 17.81	0.598
K-Sports	41.05 ± 24.81	42.81 ± 31.60	0.876
K-Quality of Life	58.88 ± 22.76	63.28 ± 26.46	0.557
KSS	84.37 ± 11.63	86.31 ± 11.05	0.551
KSS-F	72.89 ± 18.66	78.75 ± 16.21	0.290
Varus 0°	1.42 ± 0.51	1.56 ± 0.98	0.991
Valgus 0°	1.47 ± 0.84	1.66 ± 1.12	0.544
Varus 30°	3.21 ± 1.23	3.78 ± 2.09	0.315
Valgus 30°	2.79 ± 2.10	2.91 ± 2.19	0.770
Varus 60°	3.84 ± 1.89	5.09 ± 2.58	0.069
Valgus 60°	3.11 ± 2.26	3.81 ± 2.42	0.177
Varus 90°	2.84 ± 1.50	3.56 ± 1.95	0.157
Valgus 90°	2.42 ± 1.95	3.13 ± 2.31	0.267
ROM Max	112.89 ± 7.69	113.28 ± 8.95	0.710

## Data Availability

The datasets used and/or analyzed during the current study are available from the corresponding author on reasonable request.
